# Seasonal productivity of the equatorial Atlantic shaped by distinct wind-driven processes

**DOI:** 10.1038/s41561-024-01609-9

**Published:** 2025-01-06

**Authors:** Peter Brandt, Mareike Körner, James N. Moum, Marisa Roch, Ajit Subramaniam, Rena Czeschel, Gerd Krahmann, Marcus Dengler, Rainer Kiko

**Affiliations:** 1 GEOMAR Helmholtz Centre for Ocean Research Kiel, Kiel, Germany; 2 Faculty of Mathematics and Natural Sciences, Kiel University, Kiel, Germany; 3 College of Earth, Ocean, and Atmospheric Sciences, Oregon State University, Corvallis, OR USA; 4 Lamont-Doherty Earth Observatory, Columbia University, Palisades, NY USA; 5 Laboratoire d’Océanographie de Villefranche, Villefranche-sur-Mer, France; 6 Present Address: College of Earth, Ocean, and Atmospheric Sciences, Oregon State University, Corvallis, OR USA

**Keywords:** Physical oceanography, Element cycles

## Abstract

The eastern equatorial Atlantic hosts a productive marine ecosystem that depends on upward supply of nitrate, the primary limiting nutrient in this region. The annual productivity peak, indicated by elevated surface chlorophyll levels, occurs in the Northern Hemisphere summer, roughly coinciding with strengthened easterly winds. For enhanced productivity in the equatorial Atlantic, nitrate-rich water must rise into the turbulent layer above the Equatorial Undercurrent. Using data from two trans-Atlantic equatorial surveys, along with extended time series from equatorial moorings, we demonstrate how three independent wind-driven processes shape the seasonality of equatorial Atlantic productivity: (1) the nitracline shoals in response to intensifying easterly winds; (2) the depth of the Equatorial Undercurrent core, defined by maximum eastward velocity, is controlled by an annual oscillation of basin-scale standing equatorial waves; and (3) mixing intensity in the shear zone above the Equatorial Undercurrent core is governed by local and instantaneous winds. The interplay of these three mechanisms shapes a unique seasonal cycle of nutrient supply and productivity in the equatorial Atlantic, with a productivity minimum in April due to a shallow Equatorial Undercurrent and a productivity maximum in July resulting from a shallow nitracline coupled with enhanced mixing.

## Abstract (web summary)

The seasonal timing of peak primary productivity in the eastern equatorial Atlantic is a result of wind-driven processes coinciding with increased surface nitrate supply, according to transect and mooring observations.

## Main

**Fig. 1:**
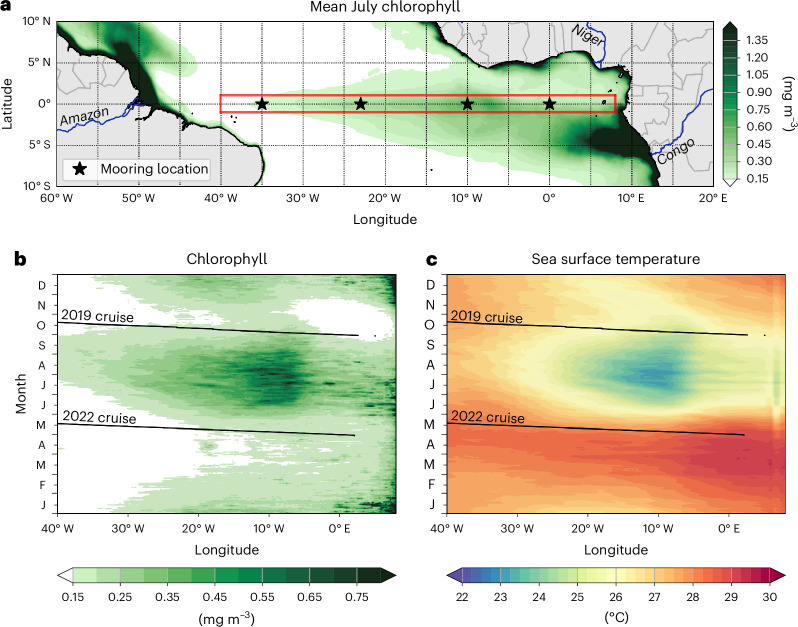
Chlorophyll and SST along with the conducted measurement programme in the equatorial Atlantic. **a**–**c**, Shown are climatological July chlorophyll in the tropical Atlantic (**a**) and the climatological seasonal cycles along the equator (meridional averaged in the red box, shown in **a**) of chlorophyll (**b**) and SST (**c**). Also included are mooring locations along the equator (**a**) and in tracks of two trans-Atlantic equatorial cruises (**b**,**c**), *R/V Meteor* cruises M158 from 29 September to 22 October 2019 and M181 from 30 April to 20 May 2022. The open ocean equatorial chlorophyll distribution that is strongly enhanced in the centre of the cold tongue at about 0°, 10° W during boreal summer is the main topic of the present study. Note the enhanced chlorophyll concentrations associated with the outflow of the Amazon and Congo rivers. The maps were created using the Python packages matplotlib (version 3.8.4)^48^ and basemap (version 1.4.1; https://matplotlib.org/basemap/stable/).

**Extended Data Fig. 1:**
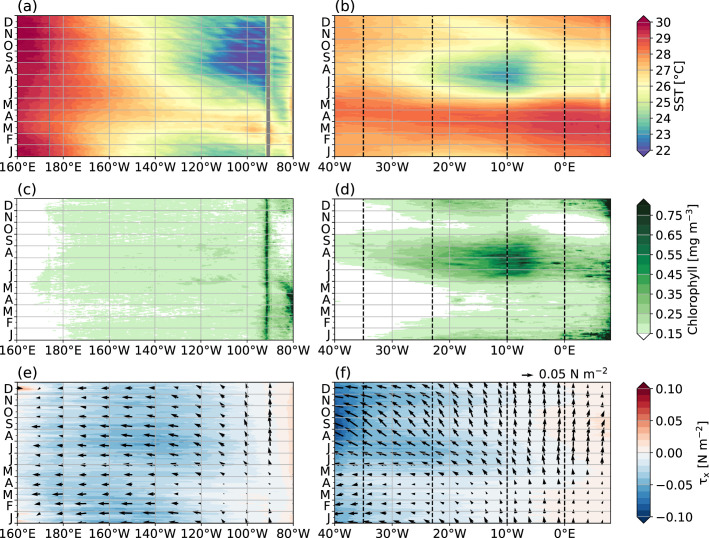
Seasonal cycle of SST, chlorophyll and wind in the equatorial Pacific and Atlantic. Climatological seasonal cycle of SST (**a, b**), chlorophyll (**c, d**), and zonal wind stress (**e, f**) as function of longitude along the equator and month of the year for the Pacific (a,c,e) and the Atlantic (b,d,f). Arrows in (e,f) mark amplitude and direction of wind stress (arrows pointing to the left mark easterly winds, arrows pointing upward mark southerly winds). Data are averaged between 1° S and 1° N. Vertical dashed lines mark the positions of PIRATA buoys in the Atlantic from which data were used for the present study. Note the different x-axis scaling for the Pacific and Atlantic. The Galápagos Islands are located at about 90° W in the Pacific and São Tomé at about 6°30’ E in the Atlantic.

**Extended Data Fig. 2:**
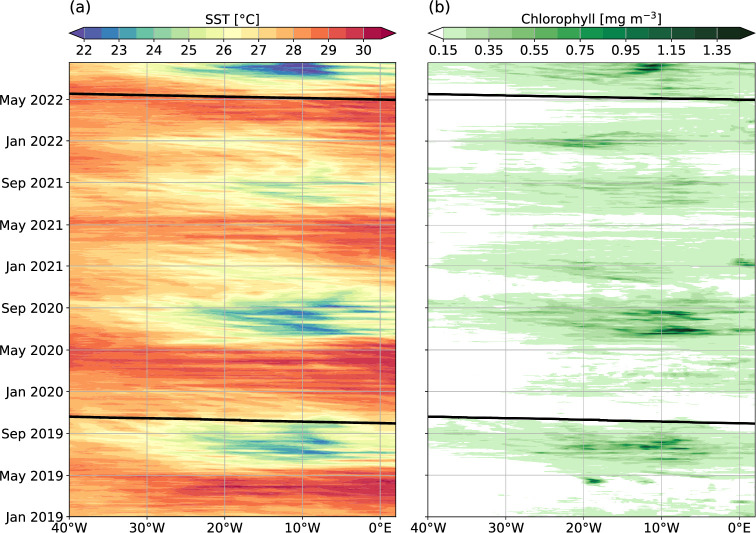
Year-to-year variability of SST and chlorophyll in the equatorial Atlantic. Hovmoeller diagrams of SST (**a**) and chlorophyll (**b**) as function of longitude along the Atlantic equator and time with tracks of the two trans-Atlantic equatorial cruises (black lines) included. Data are averaged between 1° S and 1° N.

The equatorial band is among the areas of the global ocean with the highest primary production^1^. Phytoplankton photosynthesis and organic matter production fuel productive marine ecosystems in the eastern equatorial Atlantic and Pacific^2,3^, supporting top predators such as economically important tropical tuna species^4,5^. These regions undergo strong seasonal^6^ and year-to-year variability^7^ closely related to the sea surface cooling associated with the development of equatorial cold tongues in both basins (Fig. 1 and Extended Data Figs. 1 and 2). Seasonal variations of the equatorial sea surface temperature (SST) are large in the Atlantic and dominate over interannual variability^8^. The equatorial Atlantic cold tongue seasonally develops through sea surface cooling of up to 7 °C (ref. ^9^) in response to the strengthening of equatorial easterlies during early boreal summer, driven by the northward migration of the Intertropical Convergence Zone^8^. Enhanced westward wind stress results in upwelling of colder thermocline waters in the eastern basin^10^. Sea surface cooling during the onset of the cold tongue is largely explained by the combination of thermocline shallowing and turbulence-enhanced mixing^11,12^. Forced ocean model simulations show that the upward supply of nutrients relies on the same physical mechanisms as the downward flux of heat and that the 20 °C isotherm is an excellent proxy for the nitracline, confirming similarities between upper ocean heat and nitrate distribution^13^. Whereas previous explanations of the seasonal productivity peak in the eastern equatorial Atlantic emphasized the upward movement of the nitracline^2,13,14^, here we argue that the interplay of three independent wind-driven processes shapes the seasonal chlorophyll cycle.

Surface winds force a range of phenomena in the equatorial Atlantic, including a mean wind-driven circulation composed of a westward surface flow and an eastward Equatorial Undercurrent (EUC) at thermocline level^15,16^. Three wind-forced phenomena directly contribute to the seasonality of mixing and upward nitrate flux in the Atlantic. First, the seasonal strengthening of westward wind stress along the equator drives warm surface water westward. Thereby, sea level increases to the west and the thermocline (often used as a measure of the nitracline) slopes upward to the east^10,17^. Second, whereas the EUC adjusts to zonal pressure gradient changes associated with the sea level slope^15^, equatorially trapped waves are generated by seasonally oscillating zonal winds. They propagate east- and westward, reflect at the boundaries and eventually form resonant equatorial basin oscillations^18,19^. Finally, local (in space and time) wind events initiate mixing of stratified waters in the shear layer beneath the mixed layer and above the core of the EUC. This layer is also referred to as the deep cycle layer due to presence of deep cycle turbulence that is tied to the diurnal cycle of solar heat flux^20–22^.

Here we use data from two trans-Atlantic equatorial cruises conducted at different phases of the seasonal cycle (Fig. 1), to quantify nitrate distribution and upward nitrate fluxes associated with vertical mixing. In addition, we use data from a comprehensive set of hydrographic, current and microstructure data from equatorial moorings (Fig. 1), shipboard measurements (Supplementary Table 1), Argo floats and satellites to elucidate the characteristic seasonal cycle of stratification, shear and mixing in the equatorial Atlantic. This allows us to determine the phasing of the three phenomena that set up conditions for and timing of enhanced nitrate supply by mixing and subsequent elevated productivity.

## Nitrate supply by mixing during trans-Atlantic crossings

**Fig. 2:**
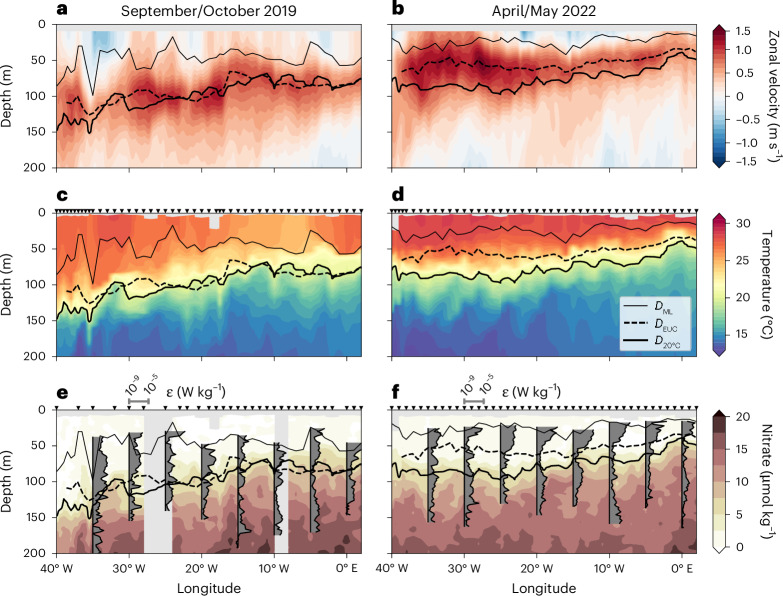
Velocity, temperature, nitrate and mixing during two trans-Atlantic crossings. **a**–**f**, Shown are zonal velocity (**a**,**b**), temperature (**c**,**d)** and nitrate (**e**,**f**) taken during *R/V Meteor* cruises M158 from 29 September to 22 October 2019 (**a**,**c**,**e**) and M181 from 30 April to 20 May 2022 (**b**,**d**,**f**). The mixed layer depth, ${D}_{\mathrm{ML}}$, is marked by thin solid lines, the EUC core depth, ${D}_{\mathrm{EUC}}$, by thick dashed lines and the depth of the 20 °C isotherm, ${D}_{20^\circ\mathrm{C}}$, as a proxy for the nitracline by thick solid lines. Also included in **e** and **f** are mean profiles of the turbulence dissipation rate, $\varepsilon$ (grey-filled areas; vertical black lines mark 10^−9 ^W kg^−1^; scale at the top of the panels). Profiles are derived by averaging all values below the mixed layer within intervals of 5° longitude. Black triangles at the top of **c** and **d** and **e** and **f** mark locations of CTD and microstructure stations, respectively.

To study seasonal variations of temperature and chlorophyll in the equatorial Atlantic^6^, we conducted two trans-Atlantic equatorial cruises from Africa to South America in September/October 2019 and April/May 2022 (Figs. 1 and 2 and Extended Data Fig. 2). The periods of both cruises were outside of major climatic events of the equatorial Atlantic. The cruise in 2019 was before the start of the major warm event peaking in January 2020^23^ and the cruise in 2022 after the Atlantic Niño peaking in July 2021^24^. Both cruises were timed to cover the two seasonal maxima of EUC core velocity^25^, which was occasionally above 1 m s^−1^. The EUC core was shallow throughout the basin (mostly above 60 m) in Apr/May and much deeper in Sep/Oct (Fig. 2a,b), when it generally shoaled towards the east.

Strengthening easterly winds in May force the development of the equatorial Atlantic cold tongue in the central and eastern equatorial Atlantic^9,26^. Here the seasonal minimum of SST is observed during July and August (Fig. 1c). Our cruises were carried out before the development of the equatorial Atlantic cold tongue (Apr/May 2022) and after its demise (Sep/Oct 2019; Extended Data Fig. 2) although remnants of the cold tongue were still visible, indicated by relatively low near-surface temperatures at 5–10° W in Sep/Oct (Fig. 2c). The 20 °C isotherm, that here is used as a proxy for both thermocline and nitracline^13^, was relatively flat in Apr/May (Fig. 2d,f). The EUC core defined by maximum eastward velocity at the equator does not follow the thermocline throughout the year. While in Sep/Oct, the EUC core was approximately aligned with the 20 °C isotherm (Fig. 2e), the EUC core was above it in Apr/May (Fig. 2f). Turbulence dissipation rate, $\varepsilon$ (W kg^−1^), a measure of the strength of turbulence, was derived using a shipboard microstructure profiler (Methods). During both cruises, $\varepsilon$ generally shows high values in the shear zone above the EUC core and low values in its core (Fig. 2e,f).

**Fig. 3:**
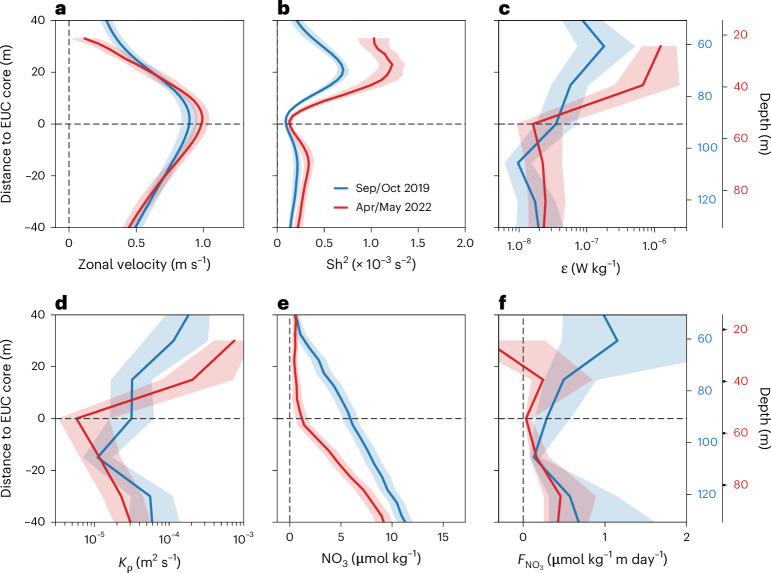
Mixing and upward nitrate flux during two trans-Atlantic crossings. Shown are different properties averaged between 35° W and 2° E relative to the core depth of the EUC in Sep/Oct 2019 (blue) and Apr/May 2022 (red). **a**–**f**, Shown are mean zonal velocity (**a**), mean squared shear, ${\mathrm{Sh}}^{2}$ (**b**), mean turbulence dissipation rate, $\varepsilon$ (**c**), mean turbulent diffusivity, ${K}_{\rho }$ (**d**), mean nitrate concentration, ${\mathrm{NO}}_{3}$ (**e**), and mean upward diffusive nitrate flux, ${F}_{{\mathrm{NO}}_{3}}$ (**f**). Absolute depth axes for the respective cruises are given on the right. Shadings mark the 95% confidence interval (Methods). Note that the almost constant nitrate gradient in Sep/Oct is due to the nitracline fluctuating around the EUC core depth in the individual profiles along the equator (Fig. 2e).

Zonal velocity profiles, averaged along the equator between 35° W and 2° E with respect to EUC core depth, reveal strong eastward flows of the EUC during both cruises with maximum velocity of 1.0 ± 0.2 m s^−1^ (mean ± one standard deviation) at its core depth of 53 ± 8 m in Apr/May and of 0.8 ± 0.1 m s^−1^ at 91 ± 13 m depth in Sep/Oct (Fig. 3a). Whereas intraseasonal meridional velocity fluctuations associated with tropical instability and wind-forced Yanai waves contribute to the vertical shear of horizontal velocity^27–29^, their effect is small^30^. The vertical distribution of squared shear, ${\mathrm{Sh}}^{2}$ (s^−2^), often linked to elevated turbulence production (Methods), shows a clear minimum at the EUC core during both cruises, thus demonstrating the dominance of zonal compared to meridional shear (Fig. 3b). Above the EUC core, shear is large with maxima approximately 20 m above the EUC core during both cruises. Stronger shear was found above the shallower EUC in Apr/May than above the deeper EUC in Sep/Oct. Mean values of $\varepsilon$ were higher above the EUC core in Apr/May (Fig. 3c).

The diapycnal diffusivity, ${K}_{\rho }$ (m^2^ s^−1^) (derived from $\varepsilon$; Methods), was large both above and below the EUC core. The EUC core, which shows a minimum of ${K}_{\rho }$, acts as a barrier for vertical diffusive fluxes. Above the EUC core, values of ${K}_{\rho }$ were higher in Apr/May than in Sep/Oct (Fig. 3d). However, the upward nitrate flux, which is the product of diapycnal diffusivity and nitrate gradient, was higher in Sep/Oct due to an enhanced nitrate gradient.

## Moored time series show seasonally elevated nitracline

**Fig. 4:**
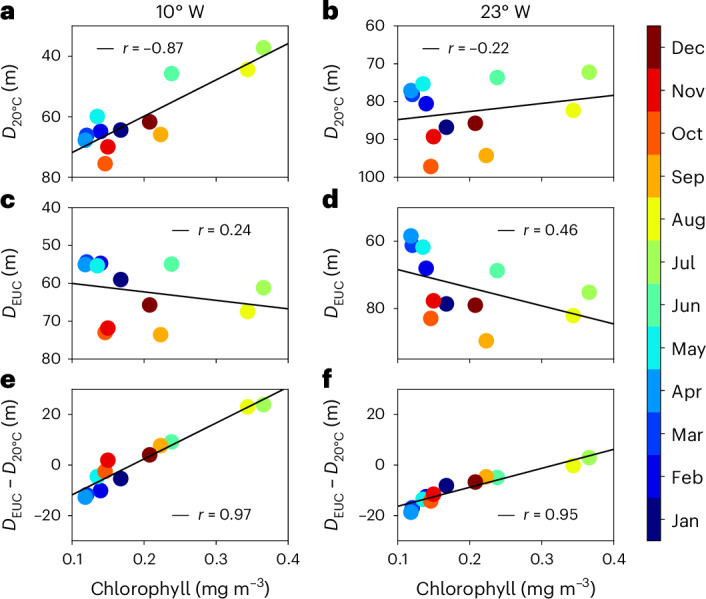
Climatological dependence of chlorophyll on nitracline and EUC core depths. **a**–**f**, Shown are scatter plots of 20 °C isotherm (or nitracline) depth, ${D}_{20^\circ\mathrm{C}}$ (**a**,**b**), EUC core depth, ${D}_{\mathrm{EUC}}$ (**c**,**d**), and their difference, ${{D}_{\mathrm{EUC}}-D}_{20^\circ\mathrm{C}}$ (**e**,**f**), versus chlorophyll for the climatological seasonal cycle at 0°, 10° W (**a**,**c**,**e**) and 0°, 23° W (**b**,**d**,**f**). Chlorophyll is averaged in the box 1° S–1° N, 30° W–0°. Also included are linear regression lines and the corresponding Pearson correlation coefficient, $r$.

**Fig. 5:**
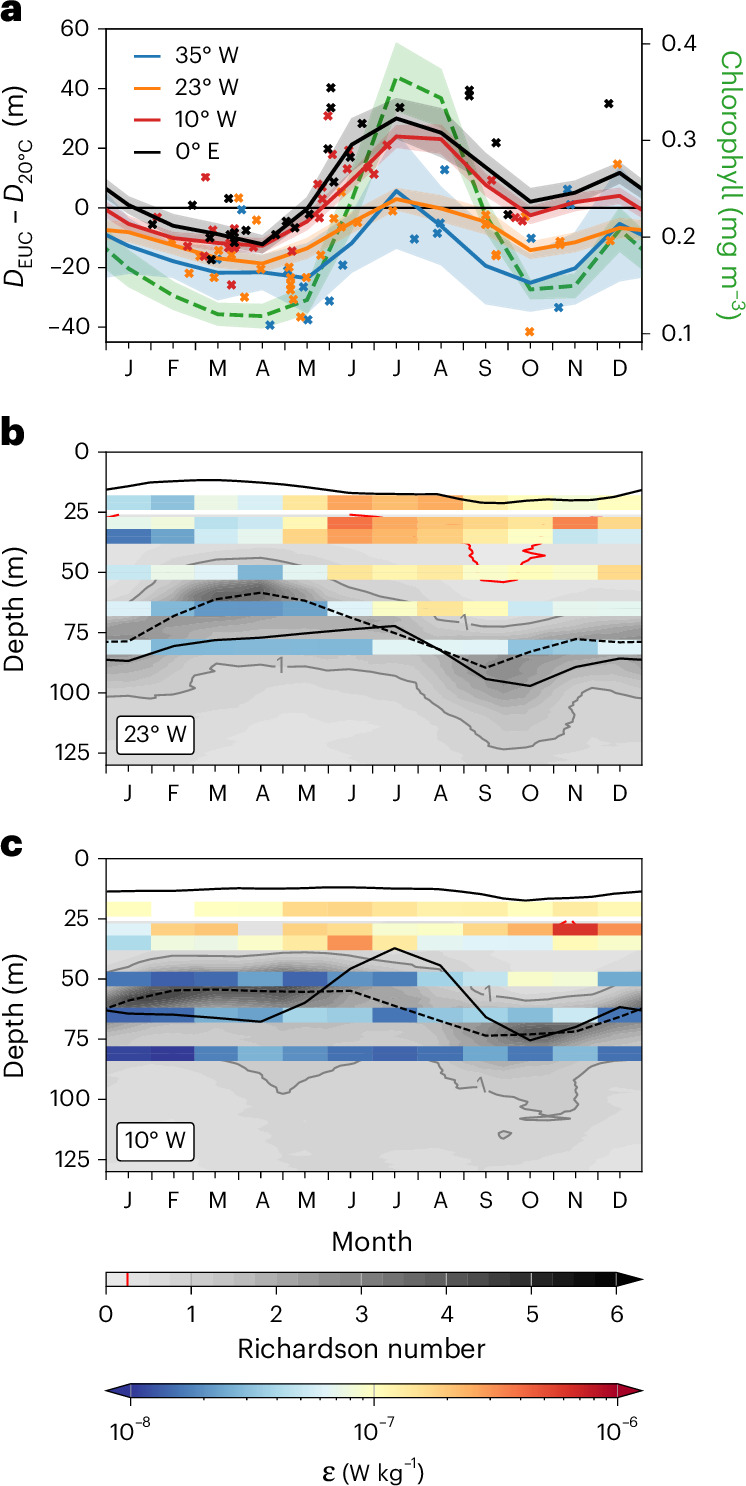
Seasonal cycle of chlorophyll, nitracline depth, EUC core depth and mixing. **a**–**c**, Shown are the seasonal cycles of the depth difference, ${D}_{\mathrm{EUC}}-{D}_{20^\circ\mathrm{C}}$, at the locations of equatorial PIRATA buoys from moored observations (solid lines represent mean values; shadings mark standard errors) and from shipboard measurements (crosses) (**a**) and the seasonal cycles at 23° W (**b**) and 10° W (**c**) of ${D}_{\mathrm{EUC}}$ (dashed lines), ${D}_{20^\circ\mathrm{C}}$ (lower black solid line). Included in **a** is chlorophyll averaged between 1° S and 1° N, 30° W and 0° (green dashed line with shaded standard error, right axis). Also included in **b** and **c** are mixed layer depth, ${D}_{\mathrm{ML}}$ (upper black solid line), Richardson number, $\mathrm{Ri}$ (grey scale with grey contour lines for $\mathrm{Ri}=1$ and red contour lines for $\mathrm{Ri}=0.25$), and turbulence dissipation rate, $\varepsilon$ (coloured bars). The depth of the 20 °C isotherm, ${D}_{20^\circ\mathrm{C}}$, is a proxy for the nitracline.

**Extended Data Fig. 3:**
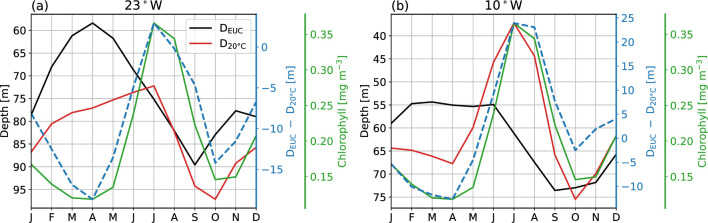
Seasonal cycle of nitracline and Equatorial Undercurrent (EUC) core depths and chlorophyll at 23° W and 10° W. Shown are of EUC core depth, ${D}_{{EUC}}$ (black lines, left axes), the depth of the 20 °C isotherm, ${D}_{20^\circ C}$, as a proxy of the nitracline (red lines, left axes), and the depth difference, ${D}_{{EUC}}-{D}_{20^\circ C}$ (dashed blue lines, first right axes), at 0°, 23° W (**a**) and 0°, 10° W (**b**) from moored observations. Also included in both panels is chlorophyll averaged between 1° S-1° N, 30° W-0° (green, second right axis).

**Extended Data Fig. 4:**
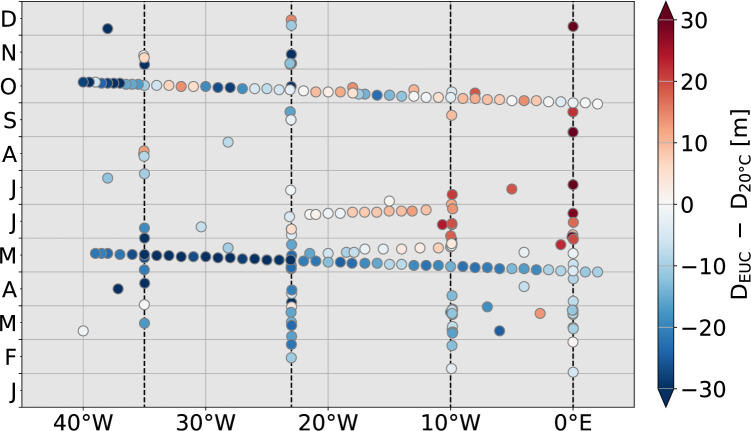
Seasonal cycle of the depth difference between Equatorial Undercurrent (EUC) core and nitracline. Difference between the EUC core depth, ${D}_{{EUC}}$, and the 20 °C isotherm depth, ${D}_{20^\circ C}$, as a proxy of the nitracline as function of months and longitude. Here, shipboard measurements listed in Supplementary Table 1 are used.

The full seasonal cycle of the depth difference between EUC core and 20 °C isotherm (and nitracline) was estimated from multi-year moored data (Supplementary Table 2)^31^. Within the seasonal cycle, high chlorophyll concentrations in the equatorial Atlantic are observed when there is a shallow nitracline at 10° W and 23° W (Extended Data Fig. 3). However, chlorophyll shows a stronger correlation with the depth difference between EUC core and nitracline than with either parameter independently (Fig. 4 and Extended Data Fig. 3). In the western and central Atlantic (35° W and 23° W), the EUC core is almost always shallower than the nitracline indicating both weak eastward advection of nitrate within the EUC and small upward nitrate flux throughout the year (Fig. 5a). In the eastern basin (10° W and 0° E), the nitracline is seasonally located above the EUC core. Largest depth differences are found in June to August, corresponding to the season with maximum productivity^6^. High-resolution profile data from research cruises (Supplementary Table 1) confirm the general behaviour of relative positions of nitracline and EUC core, with the nitracline being above the EUC core in the eastern basin from boreal summer to early winter and below the EUC core in late boreal winter and spring throughout the basin (Fig. 5a and Extended Data Fig. 4). With a minimal nitrate gradient above the EUC core, the upward nitrate flux towards the mixed layer is strongly reduced during the latter period. Occasional instances throughout the year and across the basin when the nitracline moves into the shear zone above the EUC core probably result in localized events of upward nitrate flux and enhanced productivity (Fig. 5a and Extended Data Fig. 4).

A monthly climatology from *χ*pod microstructure sensors^32^ mounted on PIRATA moorings^31^ at the equator at 23° W and 10° W shows $\varepsilon$ to be generally enhanced above the EUC core (Fig. 5b,c). Values are small at the beginning of the year and increase in May with the strengthening of equatorial easterlies. At greater depth, high values of $\varepsilon$ are found during the second half of the year at 23° W (Fig. 5b) and in October and November at 10° W (Fig. 5c), corresponding to a deep EUC core. The regions and periods of strongly enhanced $\varepsilon$ near the mixed layer roughly agree with those of Richardson number < 0.25 indicating growing instabilities and enhanced turbulence by shear production (Methods)^21,33^. High values of $\varepsilon$ are found close to the nitracline in June and July at 10° W suggesting a peak in upward nitrate flux towards the mixed layer during that period.

## Sequence of processes enhances primary productivity

In the equatorial Atlantic cold tongue, the synchronization of three different wind-driven physical processes, (1) upwelling of the nitracline, (2) deepening of the EUC and (3) enhanced turbulence in the deep cycle layer, results in peak phytoplankton productivity and biomass during boreal summer. Upwelling of the thermocline, hence nitracline, is first due to local meridional Ekman divergence associated with the strengthening of local easterlies at the equator and secondly due to zonal mass redistribution by wind-forced equatorial waves^10,17^. In the eastern Atlantic, the second mechanism dominates^10^. Whereas these mechanisms adequately explain the seasonal vertical migration of the thermocline, they do not account for the upward and downward movement of the EUC core. Instead, this movement is independently determined in the Atlantic (but not in the Pacific) by an annual oscillation caused by eastward-propagating equatorial Kelvin and westward-propagating Rossby waves. These waves, forced by seasonally oscillating zonal winds, form resonant equatorial basin modes^18,34^. Waves of the second baroclinic mode are resonant at the semiannual cycle^35^ and slower waves of the fourth baroclinic mode are resonant at the annual cycle^34^. The fourth baroclinic mode has strongest velocity amplitudes in the upper 250 m with a zero-crossing at approximately 65 m indicating reversed zonal flow above and below that depth, in turn causing an annual cycle of apparent upward and downward movement of the EUC core throughout the basin^34^. Due to the basin mode, the EUC core is thus found at different isotherms or isopycnals during its vertical migration.

**Extended Data Fig. 5:**
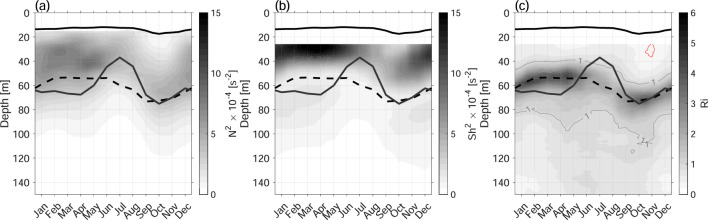
Mean seasonal cycle of different mixing parameters at the equator at 10° W. Shown are squared Brunt-Väisälä frequency, ${N}^{2}$ (**a**), squared shear, ${{Sh}}^{2}$ (**b**), and Richardson Number, ${Ri}$ (**c**). Upper solid black line marks the mixed layer depth, the lower solid black line the 20 °C isotherm depth, ${D}_{20^\circ C}$, as a proxy of the nitracline, and the dashed line the Equatorial Undercurrent (EUC) core depth, ${D}_{{EUC}}$. Also shown in (c) is the *Ri* = 0.25 contour (red line).

**Extended Data Fig. 6:**
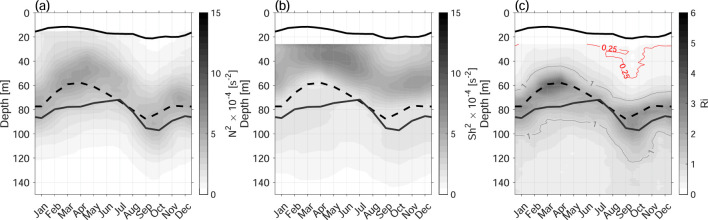
Mean seasonal cycle of different mixing parameters at the equator at 23° W. Shown are squared Brunt-Väisälä frequency, ${N}^{2}$ (**a**), squared shear, ${{Sh}}^{2}$ (**b**), and Richardson Number, ${Ri}$ (**c**). Upper solid black line marks the mixed layer depth, the lower solid black line the 20 °C isotherm depth, ${D}_{20^\circ C}$, as a proxy of the nitracline, and the dashed line the Equatorial Undercurrent (EUC) core depth, ${D}_{{EUC}}$. Also shown in (c) is the ${Ri}=0.25$ contour (red line).

Besides the wind-forced vertical displacement of nitracline and EUC core, upward nitrate flux towards the mixed layer is due to turbulent mixing^13^ associated with the vertical shear of horizontal velocity^27,28^. The largest shear is generally found in the deep cycle layer between mixed layer and EUC core (Extended Data Figs. 5 and 6). Within this layer the turbulence undergoes a diurnal cycle^20^. Turbulence in the deep cycle layer is enhanced compared to low values in the EUC core and depends on the local wind stress^21,22^. Thus, while there is strong shear in the equatorial Atlantic during boreal spring, when the EUC core is shallow, weak winds result in reduced deep cycle turbulence. With the strengthening of the winds in May turbulence increases and peaks in June contemporaneous with the shallowing of the nitracline (Fig. 5). The seasonal cycle of upward nitrate flux and productivity in the Atlantic can hence be explained by the vertical migration of the nitracline into the seasonally varying deep cycle layer.

**Fig. 6:**
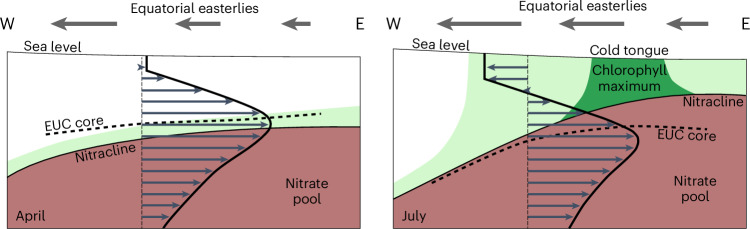
Schematic representation of the two seasonal extremes of equatorial Atlantic nitrate supply and productivity. Shown are the mean conditions in the upper 150 m during the warm (April) and cold (July) seasons. In April, the nitracline is located below the shallow EUC core, resulting in no upward mixing of nitrate. A deep chlorophyll maximum is maintained at or below the EUC core. With the strengthening of equatorial easterlies, the nitracline in the eastern equatorial Atlantic moves upward, reaching its shallowest position in July. The EUC core independently moves downward due to the presence of the annual resonant basin mode superimposed on the mean EUC. Synchronously enhanced mixing in the shear zone between surface mixed layer and EUC core, driven by increased local winds, promotes upward nitrate supply, thereby enhancing productivity up to the surface.

The results from our observational study are summarized in two schematics representing the two extremes of the seasonal cycle in the equatorial Atlantic, the low-productivity season in April and the high-productivity season in July (Fig. 6). In April easterly winds are weak, sea level and thermocline are relatively flat and there is no westward wind-driven surface flow in mid-basin. However, the EUC core shows maximum velocity and is shallow throughout the basin. Due to the shallow EUC core the nitracline is located well below the EUC core across the basin and low-nitrate waters are advected eastward by the EUC. High mixing in the shear zone above the EUC core thus occurs in warm, low-nitrate waters (Fig. 6a). This period corresponds to the period of lowest near-surface productivity in the equatorial Atlantic^6^.

In July, strong easterly winds cause sea level to rise towards the west. Consequently, the thermocline deepens in the western basin and shallows in the east^10^. Strong equatorial easterlies are associated with an energetic westward surface flow that contributes to enhanced shear below. The EUC core velocity is small in July^36^ and the EUC core deepens from its shallow phase in boreal spring to its deep phase in boreal autumn (Fig. 5b,c). With the upward movement of the nitracline and downward movement of the EUC core, the nitracline moves into the turbulent deep cycle layer above the EUC core. Coincident strong mixing (Fig. 5) causes increased upward nitrate flux. This period is followed by the period of highest productivity (Fig. 6b). The productivity maximum occurs near 10° W, whereas enhanced chlorophyll is found along most of the equator partly redistributed by strong surface currents (Extended Data Fig. 1).

Despite the similarities in the seasonality of upper ocean heat distribution and sea level cooling in the cold tongues of the Atlantic and Pacific, there are strong differences in chlorophyll pattern as measured from satellite (Extended Data Fig. 1). The annual cycle of chlorophyll concentration in the cold tongue of the eastern equatorial Atlantic is more pronounced than in its Pacific counterpart (Extended Data Fig. 1)^6,37,38^. A primary difference between Pacific and Atlantic productivity is the nutrient supply. Iron is the main limiting nutrient in the equatorial Pacific^39–41^ and is supplied to the eastern basin from the western boundary of the Pacific via the EUC^42,43^. It is possible that meridional shear by tropical instability and wind-forced Yanai waves plays a more important role for locally enhancing the productivity in the equatorial Pacific^44,45^ compared to the Atlantic by reducing the Richardson number, increasing turbulence and hence vertical iron flux toward the mixed layer out of the EUC core. Better understanding of the differences between eastern equatorial productivity in the Atlantic and Pacific will require dedicated studies of processes driving upward iron fluxes toward the mixed layer of the Pacific cold tongue.

Several biological and biogeochemical mechanisms could potentially impact future productivity along the Atlantic’s and Pacific’s equators. Among them are changes in the iron limitation of biological productivity, which has been shown to play an important role in the equatorial Pacific^39,42^ but could also influence the predominantly nitrate-limited equatorial Atlantic^40,46^. Differences in seasonally varying physical forcing in the two oceans may also have consequences for the future development of biological productivity in equatorial upwelling systems: whereas in general wind changes are thought to dictate the future evolution of equatorial upwelling^47^, changes in stratification in a warming world, along with their effects on shear and turbulent mixing and equatorial wave speeds and the Atlantic basin resonance are also likely to influence future productivity in the equatorial cold tongues.

## Methods

### Data collected during equatorial cruises

In this study we use data collected during two research cruises measuring along the Atlantic’s equator from Africa to South America. The measurements were carried out as part of *R/V Meteor* cruise M158 from 29 September 22 October 2019 and as part of *R/V Meteor* cruise M181 from 30 April to 20 May 2022. During both cruises a similar sampling strategy was used. Station work was carried out approximately every degree in longitude, where profiles of conductivity, temperature, depth (CTD) and nitrate were collected. Additionally, on most stations a microstructure profiler was used to measure turbulent dissipation rate. In addition to the station work, a shipboard Acoustic Doppler Current Profiler (ADCP) measured upper ocean velocity along the ship track.

The CTD profiles were obtained with a Seabird CTD and nitrate profiles using a UV spectrometer (type OPUS manufactured by TriOS). Salinity and nitrate measurements from the Seabird CTD and the OPUS were calibrated against water samples. Sample salinities were measured using Optimare Precision Salinometers and the calibration was derived following GO-SHIP Repeat Hydrography Manual recommendations^49^ resulting in an accuracy of the calibrated CTD salinities of approximately 0.003 g kg^−1^. Dissolved nitrate concentrations in the water column were derived from the observed attenuation of UV light in a 1 cm long path of seawater following the method described by Sakamoto et al.^50^. These concentrations were calibrated against nitrate concentrations measured from water samples. Samples were taken on board, frozen at −20 °C and transported to the home laboratory where they were measured using a Quaatro auto-analyser^51^. The resulting accuracy of the nitrate concentrations is approximately 1 µmol kg^−1^. During M158 (M181) 63 (59) CTD/OPUS profiles were measured along the equator. The location of the profiles can be seen in Fig. 2c,d. The OPUS sensor is sampling at a rate of 3 s. The resulting vertical resolution of nitrate measurements is 1.5 m for a CTD lowering speed of 0.5 m s^−1^ (typically in the upper 100 m) and 3 m at 1 m s^−1^ (below 100 m).

During M158, velocities along the equator were measured by a 75-kHz Teledyne RDI Ocean Surveyor ADCP mounted in the ship’s hull providing velocity distributions in the upper 650 m depth. During M181, velocities were measured by a 75-kHz Teledyne RDI Longranger ADCP installed in the moonpool of the ship providing velocity distribution in the upper 250 m depth. Both instruments provided velocities with a vertical resolution of 8 m with the shallowest measurement in 17 m depth. Velocity data averaged along the equator over one degree in longitude result in an error < 0.01 m s^−1^ following Fischer, et al.^52^.

During both equatorial cruises, ocean turbulence data were collected using a microstructure profiler manufactured by Sea & Sun Technology. The microstructure velocity shear measured by the instrument can be used to estimate the viscous rate of dissipation of turbulent kinetic energy, $\varepsilon$, a key parameter that quantifies turbulent mixing. The loosely tethered profiler was equipped with three airfoil shear sensors, an acceleration sensor, tilt sensors, a fast temperature sensor and standard CTD sensors. Its buoyancy was adjusted so that the profiler descended at a speed of 0.5–0.6 m s^−1^. The microstructure sampling strategy was the same during both cruises and included taking at least three profiles at each CTD station. During M158 (M181) 103 (160) microstructure profiles were measured at 35 (47) station along the equator (Fig. 2e,f). $\varepsilon$ was estimated from the shear probe data by integrating the wavenumber shear spectrum of overlapping 2 s intervals in a limited wavenumber range while assuming isotropy following Hummels, et al.^27^. Loss of shear variance due to the limited wavenumber range was accounted for by fitting the spectra to the universal Nasmyth spectrum^53^ and accounting for electronic filters and finite sensor size^54^ before integration. The 95% confidence limit of individual $\varepsilon$ estimates^55^ are within a factor 3 to 5. Uncertainties of average $\varepsilon$ depend predominately on the elevated temporal variability of turbulence in a certain oceanic region and are determined by bootstrapping techniques (below).

### Data collected during other cruises

In addition to data from the two trans-Atlantic equatorial cruises, we used data from various research cruises conducted in the equatorial Atlantic Ocean in the frame of different programmes (Supplementary Table 1).

### Mooring data

To study the seasonality of the depth of the 20 °C isotherm we made use of data from the PIRATA buoy array^31^. PIRATA buoys along the equator are located at the equator at 35° W, 23° W,10° W and 0° (Fig. 1a). They are equipped with several temperature sensors covering the upper 500 m, which are installed with increasing vertical spacing with increasing depth. From these data the depth of the 20 °C isotherm were derived and an uncertainty estimate provided by Foltz, et al.^56^. The 20 °C isotherm was chosen as a proxy for the nitracline partly for practical reasons due to the lack of adequate salinity data in the moored records. However, the comparison of the nitrate-temperature relation and the nitrate-density relation suggests that potential temperature, $\Theta$, is a similar good proxy for nitrate as potential density, ${\sigma }_{\Theta }$ (Supplementary Fig. 1).

The EUC core depths were calculated from current measurement conducted with ADCPs at four mooring locations along the equator at 35° W, 23° W,10° W and 0°. At all mooring locations current velocities in the depth range of the EUC were measured using moored ADCPs. Details about moorings can be found in refs. ^57,58^.

Turbulence data were acquired at two mooring locations at the equator at 10° W and 23° W using moored turbulence instruments (χpods) installed at different depths over the period April 2014 to April 2020. Details about the measurement and analysis of the turbulence signals are found in ref. ^32^ and about the χpod dataset used here in ref. ^21^.

### Argo float data

The Argo observation dataset from the period of 2006–2021^59^ was utilized to estimate mixed layer depth, ${D}_{\mathrm{ML}}$, and squared Brunt–Väisälä frequency, ${N}^{2}$. The Argo dataset consists of hydrographic profiles covering the upper 2,000 m of the water column. Following similar procedures of a recent study^60^, only profiles that pass the quality control (QC = 1) and that are part of the delayed mode adjusted data, were used within this study. Moreover, the profiles were required to reach at least 1,000 dbar and show continuous pressure profiles and measurements at more than 20 pressure levels in the upper 2,000 dbar. Profiles were not allowed to have data gaps of more than 150 dbar. In situ measurements were converted to absolute salinity, conservative temperature and potential density using TEOS-10^61^.

Argo profiles do not contain a uniform vertical resolution. Therefore, profiles of conservative temperature, absolute salinity and pressure were interpolated on an isopycnal grid following the modified Akima piecewise cubic Hermite interpolation method (makima method^62^) before any other calculations. Makima is based on an algorithm from Akima^63^, which builds up on the cubic spline and piecewise cubic Hermite interpolating polynomial. This interpolation scheme has been shown to work well for isopycnal gridding^61^. The chosen isopycnal grid has 433 levels ranging from 20.0–29.5 kg m^−3^, with increasing resolution for higher density levels following^60^.

In a next step, conservative temperature and absolute salinity were transformed on a pressure grid from 0:1:200 dbar via linear interpolation. For each profile, the grid points shallower than the corresponding profile’s mixed layer depth, were set to NaN.

The squared Brunt–Väisälä frequency was then estimated following TEOS-10^61,64^ as ${N}^{2}={g}^{2}{\rm{\rho }}\frac{{\rm{\beta }}{\rm{d}}{S}_\mathrm{A}-{\rm{\alpha }}{\rm{d}}T}{{\rm{d}}P}$, where *g* is the gravitational acceleration, β the haline contraction coefficient and ${\rm{\alpha }}$ the thermal expansion coefficient. ${\rm{d}}{S}_\mathrm{A}$ the absolute salinity difference, d*T* the conservative temperature difference and ${\rm{d}}P$ the corresponding pressure difference^65^. d*P* was defined to be a 10-dbar running window.

### Satellite data

The SST used is the OI-SST product (https://www.remss.com/measurements/sea-surface-temperature/). Chlorophyll data was obtained from Copernicus (10.48670/moi-00278). Wind data are from the ERA5 reanalysis product^66^ (10.24381/cds.adbb2d47). The seasonal cycle of SST and chlorophyll in the Pacific was obtained by de-weighting El Niño and La Niña years using the Ocean Niño Index^67^ applying the method described in Roch, et al.^60^. To calculate climatological seasonal cycles data between 1998 and 2021 was used.

### Mixed layer depth, EUC core depth and 20 °C isotherm depth

Mixed layer depth was determined from Argo float profiles following the Holte and Talley algorithm^68^. This algorithm is based on a mixture of threshold and gradient detection for mixed layer characteristics. Due to the higher vertical resolution available for CTD profiles from shipboard and microstructure measurements, the mixed layer depth for those profiles is defined as the depth at which the density deviates by 0.125 kg m^−3^ from the surface value.

The depth of the EUC core was determined from velocity data measured by ADCPs. This was done similarly for shipboard and moored velocity data. For each time step the depth of the maximum eastward velocity in the depth range between 20 and 150 m was determined. Afterwards a second-degree polynomial was fitted to the velocity data within a 32 m depth range centred around the maximum. The EUC core depth is then the depth where the polynomial has its maximum.

The 20 °C isotherm depth of the moored velocity was taken from the ePIRATA dataset provided by Foltz, et al.^56^. It is derived from moored temperature data taken at depths of 1 and 20 m; at 20-m intervals down to 140 m and at 180, 300 and 500 m. Temperature data are further vertically interpolated using nearby Argo float profiles to derive the 20 °C isotherm depth. The 20 °C isotherm depth of the shipboard data was calculated for each CTD station via linear interpolation of the temperature profile.

The depth difference between the EUC core and the 20 °C isotherm was determined for moored and shipboard data. For moored data, the seasonal cycle of the EUC core depth and the 20 °C isotherm depth was calculated individually before calculating the seasonal cycle of the depth difference. The standard error, $\mathrm{SE}$, of the depth difference between the EUC core and the 20 °C isotherm was calculated by first calculating $\mathrm{SE}$ for both variables individually and subsequently applying error propagation. $\mathrm{SE}$ is defined as $\mathrm{SE}=\,\frac{\sigma }{\sqrt{n}}$, where $\sigma$ is the standard deviation and $n$ is the number of independent samples. $n$ was determined by considering the decorrelation timescales as derived from the temporal autocorrelation defined via the e-folding scale (Supplementary Table 2). For the shipboard data, the depth difference was calculated for each CTD station conducted at the equator. The depth of the EUC at each CTD station was calculated from shipboard ADCP data taken during the period 2 h before to 2 h after the timestamp of the CTD station as given in data.

### Vertical diffusive nitrate fluxes

We calculated vertical diffusive nitrate fluxes from on-station turbulence and nitrate data collected during the two trans-Atlantic equatorial cruises (Fig. 3). We discarded all measurements within the mixed layer and 10 m below as the mixing efficiency is not well-defined in low stratified waters^69^. To estimate vertical diffusive nitrate fluxes, we averaged $\varepsilon$ in 15 m depth bins with respect to the EUC core depth. The diapycnal diffusivity, ${K}_{\rho }$, can be derived following ref. ^70^

1 $${K}_{\rho }=\frac{\Gamma \varepsilon }{{N}^{2}}$$

where $\Gamma$ is the mixing efficiency, here set to 0.2^69^, and ${N}^{2}$ the squared Brunt–Väisälä frequency calculated using the CTD profile taken at the same location. The diapycnal diffusivity is further used to calculate the vertical diffusive flux of nitrate following^71^

2 $${F}_{{\mathrm{NO}}_{3}}={K}_{\rho }\frac{\partial {\mathrm{NO}}_{3}}{\partial z}$$

### Richardson number

High mixing levels above the EUC core are typically identified by low Richardson numbers, $\mathrm{Ri}$, that relate the stabilizing effect of stratification to the production of turbulence by the vertical shear of horizontal velocities enumerated as squared shear. $\mathrm{Ri}$ smaller than 0.25 indicates growing instabilities and enhanced turbulence by shear production^21,33^. To obtain the seasonal climatology of the Richardson number, velocity shear from daily velocity data from the mooring sites and the seasonal climatology of ${N}^{2}$ from Argo observations were used. The velocity datasets were first interpolated on a 0:1:200 m grid following the makima method^62^. The upper 20 m of the velocity components, $\left[u,v\right]$, were set to NaN. The zonal and meridional shear components were then estimated by applying a 10-m running window. The squared shear, $\mathrm{S{h}}^{2}$, is given as $\mathrm{S{h}}^{2}={{u}_{z}}^{2}+{{v}_{z}}^{2}$, where $u$ and $v$ are zonal and meridional velocities, respectively, and subscript $z$ refers to their vertical derivative. In a next step, a 10-day mean was derived. With these 10-day means, the seasonal climatology of $\mathrm{S{h}}^{2}$ was determined following the same 15-day spacing as for the Argo dataset. Dividing the seasonal climatology of ${N}^{2}$ by the one of $\mathrm{S{h}}^{2}$, then yielded the seasonal climatology of the Richardson number, $\mathrm{Ri}=\frac{{N}^{2}}{{\mathrm{Sh}}^{2}}$.

### Averaging along the equator

For the different properties averaged along the equator with respect to the depth of the EUC (Fig. 3) we calculate the 95% confidence limits, $\mathrm{CL}95$, of the mean. For $\varepsilon$, $\mathrm{CL}95$ was derived by applying a bootstrap method^72^ to all $\varepsilon$ measurements within the respective depth bins. $\mathrm{CL}95$ for the eddy diffusivity, ${K}_{\rho }$, and the vertical diffusive nitrate flux, ${F}_{{\mathrm{NO}}_{3}}$, were calculated using standard error propagation following Hummels, et al.^27^. $\mathrm{CL}95$ of the zonal velocity, the squared shear, ${\mathrm{Sh}}^{2}$, and the nitrate concentration, ${\mathrm{NO}}_{3}$, were calculated by using the standard error, SE. The conversion from SE to CL95 is via $\mathrm{CL}95=\,\bar{x}\pm 1.96(\mathrm{SE})$ where $\bar{x}$ is the averaged property^27^. The SE is defined as $\mathrm{SE}=\,\frac{\sigma }{\sqrt{n}}$, where $\sigma$ is the standard deviation and $n$ is the number of independent samples. Each CTD profile was seen as an independent sample. For the continuous velocity measurements along the equator, the number of independent samples of zonal velocity and ${\mathrm{Sh}}^{2}$ were determined by considering the decorrelation length scales as derived from the spatial autocorrelation defined via the e-folding scale for each depth level (Supplementary Fig. 2).

### Climatologies

The SE of the difference between EUC core depth and 20 °C isotherm depth from mooring data was calculated similarly (Fig. 5a). Here we also first calculate the SE of both time series separately. The number of independent samples were again determined by considering the decorrelation timescales (Supplementary Table 2). The SE of the depth difference was then determined using error propagation.

For ${D}_{\mathrm{ML}}$ and ${N}^{2}$, seasonal climatologies were determined at the equator at 10° W and 23° W in the Atlantic from Argo float data. The seasonal climatologies were calculated as follows:

Step 1: At each of these two sites (10° W, 23° W) all Argo profiles within an ellipse with 3° radius in latitude and 8° radius in longitude were chosen. For the time spacing a 15-day grid (1:15:365) was applied as such that every 15 days profiles within ±45 days are chosen.

Step 2: On all data within the ellipse and within the time frame of ±45 days an interquartile range (IQR) filter was applied, which eliminates outliers outside 1.5 times the IQR above the third quartile and 1.5 times the IQR below the first quartile.

Step 3: Using a least-squares model regression on each of the four sites, the seasonal climatology was calculated by accounting for linear fits in longitude and latitude as well as for quadratic fits in latitude. Furthermore, a Gaussian distance weighting function was applied to the distance of each profile to the corresponding site with a decorrelation scale of 200 km.

The seasonal climatologies of ${D}_{\mathrm{ML}}$ and ${N}^{2}$ then contained 25 seasonal entries (that is, every 15 days) which were estimated from overlapping time periods. This ensures enough available Argo profiles to be included in the estimation of the seasonal climatologies. From the least-squared model, standard errors of the parameters are received as well.

## Online content

Any methods, additional references, Nature Portfolio reporting summaries, source data, extended data, supplementary information, acknowledgements, peer review information; details of author contributions and competing interests; and statements of data and code availability are available at 10.1038/s41561-024-01609-9.

## Supplementary information

Supplementary Information Supplementary Figs. 1 and 2 and Tables 1 and 2.

## Data Availability

Our research benefits from the following sources: (1) shipboard CTD measurements during *R/V Meteor* cruises M158 (10.1594/PANGAEA.952354) and M181 (10.1594/PANGAEA.952520), (2) shipboard ADCP measurements during *R/V Meteor* cruises M158 (10.1594/PANGAEA.952101) and M181 (10.1594/PANGAEA.956143), (3) shipboard microstructure measurements during *R/V Meteor* cruises M158 and M181 (available via Zenodo at 10.5281/zenodo.13381551 (ref. ^73^)), (4) additional shipboard measurements from previous cruises (Supplementary Table 1), (5) ePIRATA data (http://www.aoml.noaa.gov/phod/epirata/), (6) moored microstructure data (https://www.pmel.noaa.gov/gtmba/), (7) moored velocity data (10.1594/PANGAEA.946238), (8) satellite SST (https://www.remss.com/measurements/sea-surface-temperature/), (9) chlorophyll (10.48670/moi-00278), (10) zonal wind stress (10.24381/cds.adbb2d47) and (11) Argo float data (https://www.seanoe.org/data/00311/42182/#90179).

## Code availability

All necessary code for the data analysis and the preparation of the figures are available via Zenodo at 10.5281/zenodo.13381551 (ref. ^73^).
