# Supplementary material for: Seasonal productivity of the equatorial Atlantic shaped by distinct wind-driven processes
Source: Nat Geosci. 2025 Jan 6;18(1):84–90. doi: 10.1038/s41561-024-01609-9 (PMC11732751; doi:10.1038/s41561-024-01609-9)
Supplement: Supplementary file 1 — Supplementary Figs. 1 and 2 and Tables 1 and 2. [file 41561_2024_1609_MOESM1_ESM.pdf]

nature geoscience

Supplementary information https://doi.org/10.1038/s41561-024-01609-9

# Seasonal productivity of the equatorial Atlantic shaped by distinct wind-driven processes

In the format provided by the authors and unedited

## Contents

Supplementary Fig. 1. Nitrate-temperature and nitrate-density relations.

Supplementary Fig. 2. Statistical parameters from trans-Atlantic shipboard measurements.

Supplementary Table 1. Shipboard measurements.

Supplementary Table 2. Overview of moored observations.

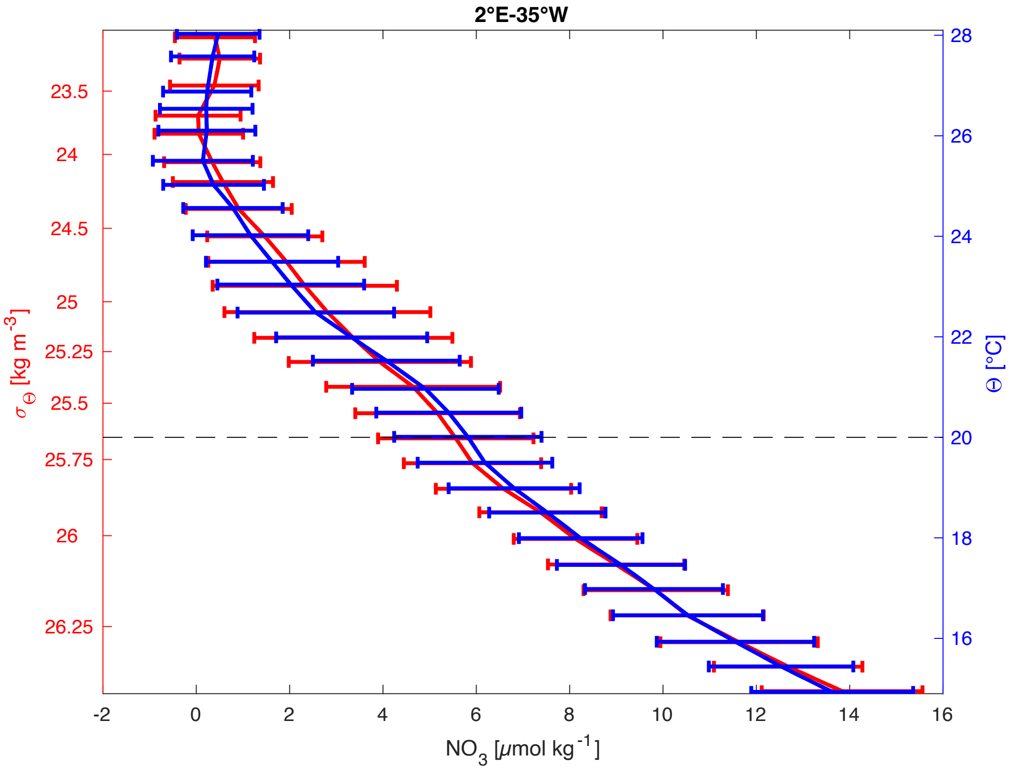


**Supplementary Fig. 1. Nitrate-temperature and nitrate-density relations.** Mean nitrate-temperature relation (blue) and mean nitrate-density relation (red) together with their standard deviations (horizontal bars) are obtained from 77 CTD/OPUS profiles taken during cruises in Sep/Oct 2019 (39 profiles) and Apr/May 2022 (38 profiles) between 35°W and 2°E (*Methods*). The profiles are assumed to be statistically independent. The comparison of both relations suggests that potential temperature, $\Theta$, is a similar good proxy for nitrate as potential density, $\sigma_\Theta$.

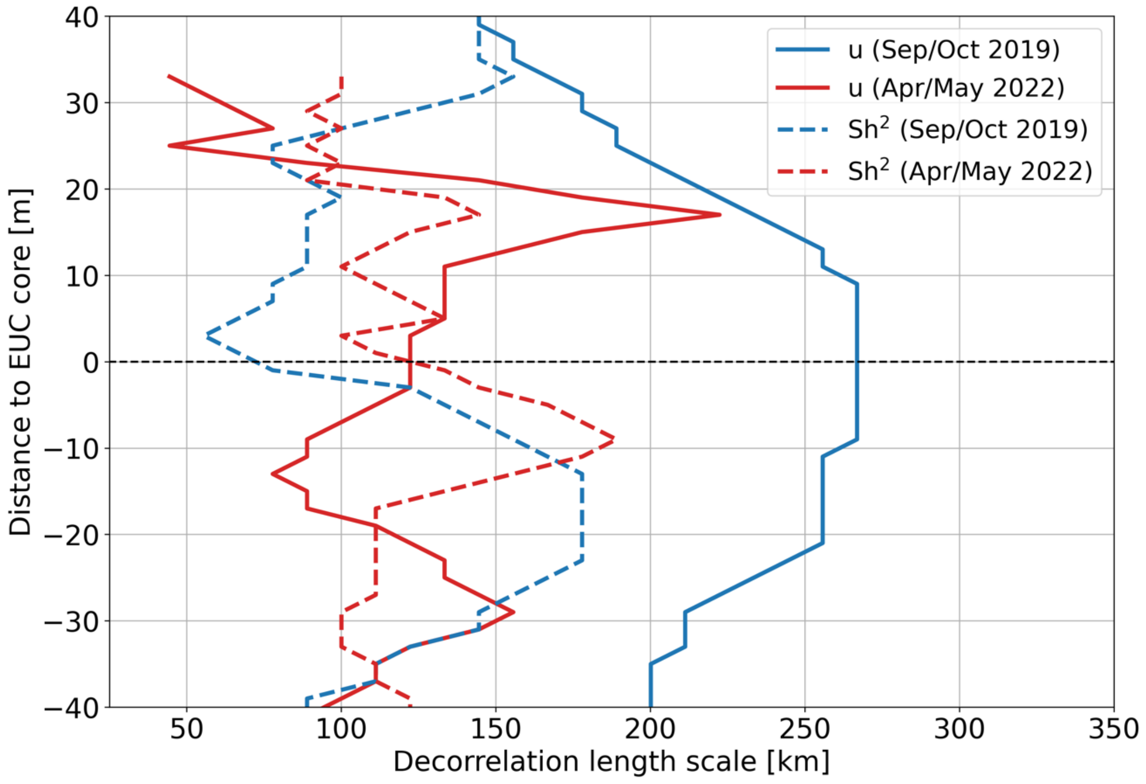


**Supplementary Fig. 2. Statistical parameters from trans-Atlantic shipboard measurements.** Decorrelation length scales of zonal velocity, $u$, and squared shear, $Sh^2$, as derived from the spatial autocorrelation of measurements along the equator.

**Supplementary Table 1.** Shipboard measurements used to identify 20°C isotherm and EUC core depths along the equator.

| Cruise | Vessel | Period | Longitude | Source |
|---|---|---|---|---|
| M16/3 | R/V Meteor | 31.05.-10.06.1991 | 35°W-30°W | https://doi.org/10.1594/PANGAEA.319324 https://doi.org/10.1594/PANGAEA.880032 |
| M22/2 | R/V Meteor | 11.04.1992 | 35°W | https://doi.org/10.1594/PANGAEA.319326 https://doi.org/10.1594/PANGAEA.880033 |
| M27/3 | R/V Meteor | 08.03.-15.03.1994 | 40°W-35°W | https://doi.org/10.1594/PANGAEA.319330 https://doi.org/10.1594/PANGAEA.290714 |
| M53/2 | R/V Meteor | 12.05.-22.05.2002 | 35°W-28°W | https://doi.org/10.1594/PANGAEA.869653 https://doi.org/10.1594/PANGAEA.952097 |
| M62/2 | R/V Meteor | 10.08.-27.08.2004 | 35°W-28°W | https://doi.org/10.1594/PANGAEA.869654 https://doi.org/10.1594/PANGAEA.952098 |
| M68/2 | R/V Meteor | 09.06.-27.06.2006 | 35°W-10°W | https://doi.org/10.1594/PANGAEA.874876 https://doi.org/10.1594/PANGAEA.787643 |
| M80/1 | R/V Meteor | 04.11.-13.11.2009 | 23°W | https://doi.org/10.1594/PANGAEA.877364 https://doi.org/10.1594/PANGAEA.834424 |
| M106/1 | R/V Meteor | 03.05.-05.05.2014 | 23°W | https://doi.org/10.1594/PANGAEA.901421 https://doi.org/10.1594/PANGAEA.869361 |
| M119/1 | R/V Meteor | 21.09.-23.09.2015 | 23°W | https://doi.org/10.1594/PANGAEA.877375 https://doi.org/10.1594/PANGAEA.860484 |
| M130/1 | R/V Meteor | 14.09.-15.09.2016 | 23°W | https://doi.org/10.1594/PANGAEA.904367 https://doi.org/10.1594/PANGAEA.904389 |
| M145/1 | R/V Meteor | 24.02.-25.02.2018 | 23°W | https://doi.org/10.1594/PANGAEA.899170 https://doi.org/10.1594/PANGAEA.904382 |
| M158 | R/V Meteor | 29.09.-19.10.2019 | 40°W-2°E | https://doi.org/10.1594/PANGAEA.952101 https://doi.org/10.1594/PANGAEA.952516 |
| M159 | R/V Meteor | 10.11.-11.11.2019 | 35°W | https://doi.org/10.1594/PANGAEA.937680 https://doi.org/10.1594/PANGAEA.952516 |
| M181 | R/V Meteor | 30.04.-18.05.2022 | 40°W-2°E | https://doi.org/10.1594/PANGAEA.956143 https://doi.org/10.1594/PANGAEA.952520 |
| MSM18/2 | R/V Maria S. Merian | 17.05.-13.06.2011 | 23°W-10°W | https://doi.org/10.1594/PANGAEA.877352 https://doi.org/10.1594/PANGAEA.783349 |
| MSM18/3 | R/V Maria S. Merian | 30.06.-18.07.2011 | 18°W-0°E | https://doi.org/10.1594/PANGAEA.783445 https://doi.org/10.1594/PANGAEA.844007 |
| MSM22/1 | R/V Maria S. Merian | 05.11.-11.11.2012 | 23°W | https://doi.org/10.1594/PANGAEA.841476 https://doi.org/10.1594/PANGAEA.830253 |
| MSM23/1 | R/V Maria S. Merian | 08.12.-09.12.2012 | 23°W | https://doi.org/10.1594/PANGAEA.842225 https://doi.org/10.1594/PANGAEA.911726 |
| SO284 | R/V Sonne | 13.07.-28.07.2021 | 35°W-23°W | https://doi.org/10.1594/PANGAEA.952519 https://doi.org/10.1594/PANGAEA.952102 |
| PIRATA-FR10 | N/O Atalante | 07.12.-14.12.2001 | 26°W-0°E | French PIRATA cruises: S-ADCP data (seanoe.org) French PIRATA cruises: CTD-O2 data (seanoe.org) |
| PIRATA-FR12 | N/O Atalante | 30.01.-13.02.2004 | 24°W-5°E | French PIRATA cruises: S-ADCP data (seanoe.org) French PIRATA cruises: CTD-O2 data (seanoe.org) |

| | | | | |
|---|---|---|---|---|
| PIRATA-FR15 | N/O Atalante | 28.05.-14.06.2006 | 10°W-0°E | French PIRATA cruises: S-ADCP data (seanoe.org)<br>French PIRATA cruises: CTD-O2 data (seanoe.org) |
| PIRATA-FR16 | N/O Antea | 24.05.-30.05.2007 | 23°W-10°W | French PIRATA cruises: S-ADCP data (seanoe.org)<br>French PIRATA cruises: CTD-O2 data (seanoe.org) |
| PIRATA-FR17 | N/O Antea | 22.06.-30.06.2007 | 10°W-0°E | French PIRATA cruises: S-ADCP data (seanoe.org)<br>French PIRATA cruises: CTD-O2 data (seanoe.org) |
| PIRATA-FR18 | N/O Antea | 04.09.-01.10.2008 | 10°W-0°E | French PIRATA cruises: S-ADCP data (seanoe.org)<br>French PIRATA cruises: CTD-O2 data (seanoe.org) |
| PIRATA-FR20 | N/O Antea | 17.09.-16.10.2010 | 23°W-0°E | French PIRATA cruises: S-ADCP data (seanoe.org)<br>French PIRATA cruises: CTD-O2 data (seanoe.org) |
| PIRATA-FR21 | N/O Suroît | 07.05.-09.06.2011 | 23°W-0°E | French PIRATA cruises: S-ADCP data (seanoe.org)<br>French PIRATA cruises: CTD-O2 data (seanoe.org) |
| PIRATA-FR22 | N/O Suroît | 25.03.-25.04.2012 | 23°W-0°E | French PIRATA cruises: S-ADCP data (seanoe.org)<br>French PIRATA cruises: CTD-O2 data (seanoe.org) |
| PIRATA-FR23 | N/O Suroît | 15.05.-03.06.2013 | 23°W-0°E | French PIRATA cruises: S-ADCP data (seanoe.org)<br>French PIRATA cruises: CTD-O2 data (seanoe.org) |
| PIRATA-FR24 | N/O Suroît | 14.04.-18.05.2014 | 23°W-0°E | French PIRATA cruises: S-ADCP data (seanoe.org)<br>French PIRATA cruises: CTD-O2 data (seanoe.org) |
| PIRATA-FR25 | N/O Thalassa | 23.03.-11.04.2015 | 23°W-0°E | French PIRATA cruises: S-ADCP data (seanoe.org)<br>French PIRATA cruises: CTD-O2 data (seanoe.org) |
| PIRATA-FR26 | N/O Thalassa | 12.03.-06.04.2016 | 23°W-0°E | French PIRATA cruises: S-ADCP data (seanoe.org)<br>French PIRATA cruises: CTD-O2 data (seanoe.org) |
| PIRATA-FR27 | N/O Thalassa | 02.03.-28.03.2017 | 23°W-0°E | French PIRATA cruises: S-ADCP data (seanoe.org)<br>French PIRATA cruises: CTD-O2 data (seanoe.org) |
| PIRATA-FR29 | N/O Thalassa | 06.03.-30.03.2019 | 23°W-0°E | French PIRATA cruises: S-ADCP data (seanoe.org)<br>French PIRATA cruises: CTD-O2 data (seanoe.org) |

| | | | | |
|---|---|---|---|---|
| PIRATA-FR30 | N/O Thalassa | 23.02.-16.03.2020 | 23°W-0°E | French PIRATA cruises: S-ADCP data (seanoe.org)<br>French PIRATA cruises: CTD-O2 data (seanoe.org) |
| PIRATA-FR31 | N/O Thalassa | 09.03.-03.04.2021 | 23°W-0°E | French PIRATA cruises: S-ADCP data (seanoe.org)<br>French PIRATA cruises: CTD-O2 data (seanoe.org) |
| PIRATA-BR04 | N/O Antares | 10.04.-06.05.2001 | 38°W-23°W | http://pirata.ccst.inpe.br/en/data-2/<br>https://www.seanoe.org/data/00696/80771/ |
| PIRATA-BR05 | N/O Antares | 29.03.-15.04.2002 | 38°W-35°W | http://pirata.ccst.inpe.br/en/data-2/<br>https://www.seanoe.org/data/00696/80771/ |
| PIRATA-BR07 | N/O Antares | 27.07.-18.08.2004 | 38°W-35°W | http://pirata.ccst.inpe.br/en/data-2/<br>https://www.seanoe.org/data/00696/80771/ |
| PIRATA-BR09 | N/O Antares | 12.11.-06.12.2006 | 38°W-35°W | http://pirata.ccst.inpe.br/en/data-2/<br>https://www.seanoe.org/data/00696/80771/ |
| PIRATA-BR10 | N/O Antares | 28.03.-21.04.2008 | 38°W-35°W | http://pirata.ccst.inpe.br/en/data-2/<br>https://www.seanoe.org/data/00696/80771/ |
| PIRATA-BR11 | N/O Antares | 21.03.-01.04.2009 | 38°W-35°W | http://pirata.ccst.inpe.br/en/data-2/<br>https://www.seanoe.org/data/00696/80771/ |
| PIRATA-BR12 | N/O Antares | 24.07.-15.08.2010 | 38°W-35°W | http://pirata.ccst.inpe.br/en/data-2/<br>https://www.seanoe.org/data/00696/80771/ |

**Supplementary Table 2.** Overview of moored observations including period of observations, days of measurements, $N$, and decorrelation time scale.

| EUC core depth | | | |
|---|---|---|---|
| | Period of observations | N [d] | Decorrelation time scale [d] |
| 35°W | Jan 2005 – Jun 2006 | 525 | 52 |
| 23°W | Dec 2001 – Jun 2023 | 6686 | 41 |
| 10°W | May 2003 – Mar 2019 | 4436 | 24 |
| 0° | Mar 2007 – Jun 2011 | 1338 | 26 |
| | | | |
| 20°C isotherm depth | | | |
| | Period of observations | N [d] | Decorrelation time scale [d] |
| 35°W | Jan 1998 – Dec 2022 | 9110 | 62 |
| 23°W | Mar 1999 – Dec 2022 | 8701 | 31 |
| 10°W | Sep 1997 – Dec 2022 | 9239 | 36 |
| 0° | Feb 1998 – Dec 2022 | 9099 | 36 |
